# Electronic Health Record Adoption and Its Effects on Healthcare Staff: A Qualitative Study of Well-Being and Workplace Stress

**DOI:** 10.3390/ijerph21111430

**Published:** 2024-10-28

**Authors:** Maria Provenzano, Nicola Cillara, Felice Curcio, Maria Orsola Pisu, Cesar Iván Avilés González, Maria Francisca Jiménez-Herrera

**Affiliations:** 1 Campus Catalunya, Universitat Rovira i Virgili, 43002 Tarragona, Spain; maria.provenz@gmail.com; 2 Department of Surgery, Ospedale Santissima Trinità, 09121 Cagliari, Italy; ncillara@gmail.com; 3 Faculty of Medicine and Surgery, University of Sassari (UNISS), 07100 Sassari, Italy; 4 Azienda Ospedaliero Universitaria, 50134 Cagliari, Italy; mpmariapisu7@gmail.com; 5 Department of Nursing, Universidad Popular del Cesar, Valledupar 200002, Colombia; cesari.avilesg@unica.it; 6 Department of Medical Sciences and Public Health, University of Cagliari, 09042 Cagliari, Italy; 7 Nursing Department, Universitat Rovira i Virgili, 43007 Tarragona, Spain; maria.jimenez@urv.cat

**Keywords:** digital patient records, healthcare workers, workplace stress, technostress

## Abstract

Adopting electronic health records (EHRs) offers improved communication and information sharing and reduces medical staff errors. Despite these potential benefits, EHR adoption often introduces new challenges for healthcare staff, including increased administrative burdens and workplace stress. This study examines the impact of EHR systems on the well-being and workplace stress of healthcare staff in a hospital setting. Using a qualitative multi-perspective research approach, 16 guideline-based interviews were conducted to explore experiences, insight, and perceptions surrounding the anticipated introduction of EHRs. Data analysis reveals a complex interplay between the perceived advantages of EHRs, such as improved data accessibility and patient safety, and the challenges related to increased workload. Based on interviewers’ perceptions, emerging themes were categorized as technostress creators or inhibitors. The findings highlight a dual impact of EHRs: while participants acknowledged improvements in patient safety and information access, they also expressed concerns about increased workload, technostress, and potential disruptions to team dynamics. This study identified two major themes: “EHR Adoption as a Double-Edged Sword” and “EHRs’ Influence on Professional Dynamics”. These findings underscore the need for organizational readiness and staff support to mitigate the negative impacts of EHRs on healthcare workers’ well-being and job satisfaction.

## 1. Introduction

The adoption of electronic health records (EHRs) has transformed healthcare systems globally, influencing both patient care and the daily experiences of healthcare staff.

Digitalization helps to increase efficiency, facilitates workflows, and represents immense potential for work organizations. EHR systems have been promoted to reduce documentation errors, improve communication and information transparency, decrease medical error, and facilitate access to patient data [1,2,3,4]. However, the transition from paper-based systems to digital platforms has not been without challenges, particularly in relation to the well-being and workplace stress experienced by healthcare workers.

**Figure 1:**
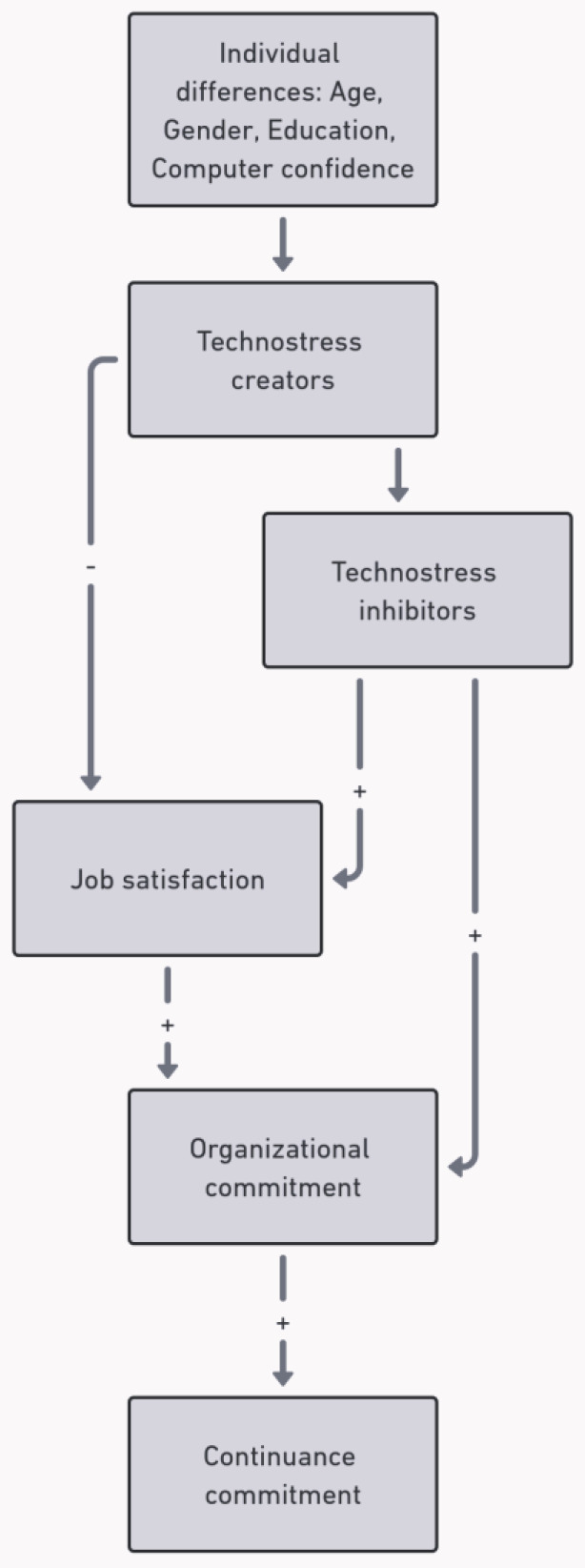
Visualization of technostress model.

Healthcare staff often suffer increased administrative burdens and workflow disruptions. Studies have found that these problems resulted in clinician frustration, caused errors in patient care, reduced interaction between patients and clinicians, and that healthcare workers experience technostress—a form of psychological strain resulting from the use of complex digital systems [5,6]. According to Ragu-Nathan et al., technostress refers to “the stress experienced by end-users in organizations as a result of their use of ICT” (Figure 1). This stress arises from the need to learn new technologies, manage system malfunctions, and cope with the increased cognitive load that EHR systems can create.

The demands for digital competences and associated changes in the role of health professionals also require a change in the perception of and attitude toward digital resources in everyday work. A significant concern is the impact of EHRs on job satisfaction and professional autonomy. Healthcare professionals, particularly physicians and nurses, report feelings of frustration as they spend more time on data entry and less time on patient care [4,7]. The rigid workflows imposed by EHR systems affect their sense of control over their clinical work, contributing to burnout [8,9,10,11].

In Italy, the push towards digitalization in the healthcare sector has been significantly accelerated by the National Recovery and Resilience Plan (Piano Nazionale di Ripresa e Resilienza, PNRR). As part of the European Union’s broader NextGenerationEU program, which aims to support EU member states in recovering from the economic and social impacts of the COVID-19 pandemic, the PNRR allocates substantial funding to modernize the healthcare system [12]. EHR implementation is a key component in creating an interoperable, nationwide EHR system that improves the efficiency and continuity of care. The PNRR has set ambitious targets to expedite the digital transformation of healthcare services, with a significant portion of the EUR 15 billion allocated to healthcare, earmarked for digital health innovations, including EHRs. These efforts aim to unify health records and enable seamless data sharing across regions and healthcare institutions, modernizing patient care and ensuring that healthcare professionals have access to entire patient care histories in real time. However, this rapid digitalization and the struggling timelines [13] established to secure financial aid have intensified workplace stress for many healthcare workers. Consequently, it is becoming increasingly important to assess how hospital digitization processes affect the well-being of healthcare staff. Identifying stressors and available resources, as well as understanding their interaction, can be a key factor of a successful transition to an EHR system.

The aim of this study is to (1) understand the expectations of healthcare staff regarding the upcoming introduction of the EHR system in a hospital setting, with a specific focus on its impact on workplace well-being, and (2) identify the stressors and resources arising from the use of digital technologies.

## 2. Materials and Methods

### 2.1. Research Team and Reflexivity

Reflexivity is a crucial aspect of establishing credibility in qualitative research [14]. Although participants’ identities were anonymized, they were professionally acquainted with the lead researcher, a female Registered Nurse working in the same outpatient department where the study was conducted. To mitigate potential bias arising from the lead researcher’s professional relationship with participants, she engaged in regular reflective discussions with the other authors. These discussions promoted transparency regarding the researchers’ perspectives and their potential influence on the study, thereby enhancing its overall trustworthiness [15].

### 2.2. Study Design

This study employs qualitative research design with a multi-perspective approach, using Interpretative Phenomenological Analysis (IPA) as the methodological framework [16]. This design aims to explore the individual and collective experiences of healthcare staff regarding the anticipated implementation of the electronic health record (EHR) system. The multi-perspective approach enables this study to capture diverse viewpoints from various roles within the healthcare team, such as nurses and physicians, to understand how EHRs may affect their work and well-being from multiple perspectives. By employing the IPA framework, this study delves deeply into how these individuals make sense of their lived experiences, perceptions, and expectations related to the EHR system.

IPA’s emphasis on subjective meaning and personal interpretation is particularly valuable for understanding how each staff member perceives the potential benefits and challenges of the new system while also considering the broader workplace context in which these experiences unfold. Consistent with Heidegger’s notion that individuals’ realities are inherently shaped by the world around them, IPA recognizes that meanings are always constructed through interactions with others. This combination of qualitative research methods allows for a rich, in-depth exploration of the emotional, practical, and relational impacts of the EHR system on healthcare staff.

This study’s findings are reported in accordance with the Consolidated Criteria for Reporting Qualitative Research (COREQ) [17], ensuring transparency and rigor in the presentation of results.

### 2.3. Sampling and Recruitment

Participants in this study were purposively recruited from different roles within the General Surgery Unit, providing a diversity of perspectives. This multi-perspective approach includes nurses, as frontline caregivers, and physicians.

In line with the theoretical framework of Interpretative Phenomenological Analysis (IPA), this study will focus on a convenient and small sample to allow an in-depth exploration of participants’ lived experiences. Participant enrollment continued until data saturation was achieved (n = 16). This sample size is deemed adequate for gathering rich, comprehensive data that capture each participant’s unique perspective. It also aligns with studies showing that saturation can be reached within a small range of interviews (9–17) [18]. Saturation is defined as the point during data collection where additional data do not provide new information to the researchers [19].

The inclusion criteria for this study include the condition that participants must be currently employed within the Unit and have direct involvement in patient care that will be affected by the implementation of the EHR system.

The participants in this study were intentionally recruited using convenience sampling to ensure a diversity of perspectives and experiences. The inclusion criteria focused on different roles in direct patient care, such as nurses and doctors, as the implementation of EHRs would directly impact both groups. Although gender was not an intentional selection criterion, a balance between male and female participants was achieved unintentionally. Furthermore, staff with varying levels of work experience, ranging from seasoned professionals to more recent hires, were included, ensuring the collection of a wide range of viewpoints on adopting EHRs.

There were no restrictions based on age or length of service for participants. The objective was to capture diverse perspectives and experiences with EHR implementation. Consequently, all employees directly involved in patient care could participate in the research. This approach allowed for a deep exploration of participants’ lived experiences, aligning with Interpretive Phenomenological Analysis (IPA) principles, which seek to understand how individuals interpret their experiences in specific contexts. Including a diverse sample in terms of age and tenure facilitated a richer and more nuanced representation of the perceptions and impact of EHRs on the well-being and workload of healthcare personnel (Table 1).

During the recruitment phase, the internal surgical ward had 17 nurses and 13 physicians. Of these, 8 nurses (ISCO code 2221) [20], and 8 medical doctors (ISCO code 2212) [20] agreed to participate in this study.

This purposive sampling, carried out by the authors MP and NC, allowed researchers to explore healthcare staff expectations, state of mind, and worries toward the imminent introduction of EHRs.

### 2.4. Data Collection and Analysis

Participants were invited in person. No one refused the interview. Data were collected through semi-structured, face-to-face, in-depth interviews. Semi-structured interviews are the most common method for data collection in IPA studies, as they elicit rich, first-person accounts of participants’ experiences with the phenomenon under investigation. The semi-structured interview format provides a consistent framework for topics to be explored while yielding rich, reliable, and comparable qualitative data [21].

The researcher and respondents were alone during the interviews.

The interviews began with a brief collection of baseline demographic information, including participants’ age, gender, years of experience, and role within the Unit. This introductory step helped contextualize their responses and provided a background to better understand the variety of perspectives. An interview guide (Appendix A) was used flexibly to foster natural conversation and encourage participants to elaborate on their perceptions, as well as to explore systematically various aspects of the main topic.

The first question was used to introduce the argument. The duration of the interviews varied, with a mean length of 27 min.

No minimum duration was set by default as the interviewer allowed participants to express themselves in complete freedom and in their own manner and style—whether concisely, descriptively, or even with digressions.

The question language was simple and clear. Field notes were also taken to capture non-verbal cues, emotional nuances, and the context of interactions that were not fully evident in the audio recordings. These notes complemented the verbatim transcripts by providing additional layers of interpretation, such as participants’ body language, tone of voice, and emotional reactions, which enriched our understanding of the lived experiences being studied. This method was chosen because it allows for a flexible yet structured approach, enabling the researcher to guide the conversation while allowing participants to expand on their unique experiences.

An informed consent form was presented to the participant at the beginning of each interview. After reading it, the researcher had a brief conversation to ensure that the participant understood the study’s purpose and the research focus.

The analysis followed a rigorous process. Verbatim transcriptions of each interview were integrated with the interviewer field notes regarding attitudes and paralinguistic features, ensuring the highest level of accuracy. The transcripts were then meticulously reviewed multiple times, allowing the researchers to become deeply immersed in the data. During this initial phase, key themes and insights began to emerge, and the researchers noted significant phrases and concepts, which laid the groundwork for subsequent analysis.

The next step involved open coding, during which the researchers systematically identified and highlighted relevant patterns, concepts, and ideas within the transcripts. This process facilitated the emergence of initial themes that reflected core concepts related to participants’ expectations, concerns, and experiences regarding the implementation of the EHR system. These themes, encapsulating the essence of each participant’s narrative, were grouped into thematic clusters based on conceptual similarities.

The interpretative phase consists of deeper reflection on these themes. The researchers contextualize participants’ experiences and insight to broader issues in healthcare, such as changes in workflow, data management challenges, and potential impacts on job satisfaction and workplace well-being. This phase aimed to go beyond surface-level descriptions to interpret the underlying meanings of participants’ responses within the context of their professional environment. The final step consists of categorizing emerging themes in technostress creator or technostress inhibitor and exploring their differences in nurses and physicians.

Consensual validation was performed between the two researchers M.P and N.C, with no disagreements emerging. This validation step helped confirm the credibility of the findings and ensured that they aligned with participants’ perspectives.

### 2.5. Ethical Consideration

This study was conducted in full compliance with the ethical guidelines set forth in the Declaration of Helsinki [22], the Italian privacy law (Decree No. 196/2003) [23], and the General Data Protection Regulation (GDPR-EU 2016/679) [24]. The study protocol received approval from the Head of the ward where the field research took place. Prior to the interviews, participants were thoroughly briefed, both orally and in writing, on the study’s objectives. Each participant signed an informed consent form, acknowledging their understanding of the study’s purpose, their voluntary participation, and their right to withdraw at any time without any adverse consequences.

Participants were assured that their anonymity and the confidentiality of the data would be protected by the authors. To safeguard privacy, each interview was assigned an alphanumeric code, ensuring there was no way to identify individual participants.

## 3. Results

Data analysis identified two overarching themes: “EHR Adoption as a Double-Edged Sword” and “EHRs’ Influence on Professional Dynamics”.

### 3.1. EHR Adoption as a Double-Edged Sword

This theme reflects the mixed emotions expressed by participants. The awareness of the initial effort and increased cognitive workload due to technological challenges and system integration issues is counterbalanced by the recognition of long-term benefits. The tension between these two elements is the core factor of the theme.

On one hand, participants recognize more efficient workflows as a long-term benefit of EHR implementation. An EHR system could be a tool that could enhance patient safety, improve data accessibility, and reduce documentation errors, particularly those stemming from handwriting issues or manual transcription (I.01, I.05, I.16). Participants emphasized that having integrated clinical records supports more informed decision-making and enhances continuity of care, especially when a patient is transferred to a different department or discharged (I.01, I.02, I.09, I.14).

On the other hand, participants are aware that the initial period will not be without difficulties, confusion, and a slowdown in work. They expressed concerns about the learning curve associated with EHR adoption, particularly for older staff members. In their point of view, the transition period would be marked by stress, pressure, and frustration (I.01, I.11, I.12). “The transition will likely slow us down initially, and I’m concerned it might remain that way for some time” (I.02). Participants feared that electronic documentation might require more time and attention, potentially adding to their already demanding workload (I.05). Many participants expressed the hope that the software would be easy to use and not introduce new inefficiencies caused by frequent glitches or unnecessarily complicated processes (I.01, I.11).

### 3.2. EHRs’ Influence on Professional Dynamics

Reshaping professional dynamics and influencing team collaboration is the second overarching theme centered on the EHR system introduction. One participant noted that having everything “written down and available to everyone” could reduce miscommunication and misunderstandings between healthcare providers (I.06). Another participant stated “We no longer have to ask for clarifications on everything; we will have things in black and white, and everyone will take responsibility for their own actions” (I.16). Participants highlighted that shared access to patient information could promote better communication between nurses and physicians, breaking down silos in care provision: “We are different component of the same patient centered care” (I.03).

While many participants believed that the EHR system could enhance communication and transparency by providing shared access to patient data, some expressed concerns that it might exacerbate existing generational divides. Younger staff members, more comfortable with technology, might find themselves frequently assisting older colleagues, which could lead to overwork, frustration, and potential power imbalances within teams (I.06, I.07, I.09, I.10, I.12). Another potential obstacle to team cohesion was identified in the disparity in EHR proficiency levels. This skill gap could create a competitive rather than collaborative environment, where certain staff members “shine” due to their technological proficiency, leaving others feeling marginalized or inadequate (I.04) (I.06).

Participants worried that the EHR system might reduce direct, face-to-face interaction between healthcare providers. One participant mentioned, “The reliance on EHR might lead to less face-to-face interaction, as staff may feel they no longer need to communicate directly if everything is documented electronically” (I.12). This could affect the quality of teamwork and contribute to a more fragmented approach to patient care.

### 3.3. Technostress Creator and Technostress Inhibitors

The implementation of electronic health records (EHRs) in healthcare settings has resulted in a range of technostress creators and inhibitors that influence the overall well-being and job satisfaction of healthcare professionals. Through data analysis, this study identifies the digital stressors and technostress inhibitors present in the sample under investigation. The multi-perspective approach enabled us to identify both the factors common to the entire sample and those specific to each professional role.

Technostress creators, or digital stressors (Table 2), arise from various challenges encountered during the adoption and use of EHR systems:

Increased Workload and Time Demands: The introduction of EHRs initially increased the workload of and time demands on healthcare staff. Participants frequently reported concerns about reduced time for direct patient care and an overall increase in administrative burdens (I.01, I.03, I.04, I.06, I.09, I.10, I.15).

Device Equipment Issues: Insufficient, obsolete, or malfunctioning device equipment was reported as a significant stressor. Technical difficulties with device functionality delayed the operation of EHR systems, increasing stress and reducing overall productivity (I.03, I.04, I.09). “The system is excellent, but I think we lack adequate technological tools to support it. We have slow computers that frequently malfunction, and we don’t have advanced technology—no tablets, no handheld device” (I.09).

User Experience of EHR: Participants noted that poor user experience, characterized by complex interfaces or non-intuitive navigation, contributed to increased frustration (I.01, I.09, I.11, I.12, I.15).

Generational and Technological Proficiency Gaps: There was a notable divide between younger and older staff members in terms of comfort and proficiency with digital technologies. Younger professionals generally adapted more quickly, while older staff expressed greater difficulties, leading to power imbalances and reliance on younger colleagues for assistance (I.01, I.03, I.04, I.06, I.09, I.10, I.12, I.13, I.14, I.15). “We are in a period where multiple generations coexist, which makes it somewhat difficult to introduce this tool. I believe it will be a bit complicated, and the benefits will probably be seen when the younger generations completely take over—not because older staff members are less capable, but because I understand that they might not be as comfortable or familiar with it. Their mindset is sometimes resistant to changes that might seem daunting or disrupt the way things have always been done. For them, it’s fine the way it is, so why change it? Sometimes, new developments are not perceived as positive, and there isn’t the same mental flexibility as there is now. I think it will be challenging to implement everything concretely” (I.15).

Low Interoperability and Paper Survivor: Interoperability refers to the ability of different digital systems, devices, or software applications to connect, communicate, and exchange data in a coordinated manner. Paper survivor refers to the persistence of paper-based documentation and record-keeping practices despite the implementation of digital systems like EHRs. The continued use of paper for certain tasks or records, even after the adoption of digital systems, can undermine the benefits of digitalization (I.08, I.09). “I’m concerned about how this new tool will communicate with all the other systems we already use. I expect that paper documentation won’t be eliminated, leading to an increased workload and double work using both digital and paper formats. I think this will create a lot of confusion and numerous different procedures that we will have to address, but entirely on our own” (I.10).

Low Confidence in the Organization and Training Quality and Support: Several participants expressed low confidence in the organization’s ability to provide adequate training and support during the EHR implementation. This lack of trust was linked to previous experiences with insufficient preparation, and inadequate technical support in everyday work (I.12, I.09, I.10, I.01, I.02). “When we have a problem with a computer that isn’t working or doesn’t connect to the servers, it takes a long time before it gets fixed. When we become completely dependent on the IT system, I hope that support will be quick and effective” (I.13).

The specifical digital stressors for physicians are as follows:

Resistance and Opposition from Part of the Team: Resistance to EHR adoption from certain team members, particularly those who were less comfortable with technology, was identified as a barrier to successful implementation. This resistance often led to reluctance in adopting new workflows and increased friction within the team (I.01, I.15). “Someone will be more involved in training the older generations, if not replacing them, or even saying, “Leave it, I’ll do it”, because in the end, it’s faster to do it yourself than to teach them when there’s no willingness to learn” (I.09). “My hope is that these challenges are genuine and not just raised to stir up unnecessary conflict within the group. Often, when faced with greater difficulties, there is a tendency to create problems rather than focus on finding solutions. That’s what worries me” (I.04).

Documentation Pressure and Legal Liability: It encompasses concerns related to the timeliness and accuracy of documentation, fear of retrospective scrutiny, and potential legal implications arising from delayed or incomplete records (I.13, I.14, I.01, I.11). “I think it will greatly increase my workload because it will require promptness. What concerns me the most is that the recording of data, especially regarding clinical notes, will demand real-time entry” (I.11).

The specifical digital stressor for nurses is as follows:

Assignment of Non-Nursing Tasks, Including Administrative Duties: Performing non-nursing duties outside their core clinical and patient care responsibilities, such as administrative duties, documentation, or IT-related troubleshooting, can diminish the professional identity of nurses. This can lead to a perceived devaluation of their role within the healthcare team (I.04, I.07, I.16, I.10).

Technostress inhibitors, or protective factors, help mitigate the negative effects of technostress and support the successful integration of EHR systems:

Increased Transparency and Accountability: The digital nature of EHR systems enhances transparency and accountability, as all actions are logged and easily traceable. This promotes a clearer understanding of patient interactions and can improve the quality of care provided by healthcare professionals (I.01, I.03, I.04, I.06, I.09, I.10, I.15).

Reduction in Errors and Increased Accuracy: Participants acknowledged that EHR systems could reduce documentation errors and improve data accuracy, especially in terms of medication administration and patient records. This improvement can enhance clinical outcomes (I.01, I.03, I.04, I.06, I.09, I.10, I.15).

The specifical technostress inhibitors for nurses are as follows:

Professional Role Enhancement: The introduction of EHRs was seen as an opportunity to elevate the professional roles of nurses. Recognizing the importance of both professions within the EHR system fostered a sense of validation and professional recognition (I.03, I.06, I.16). “The expectation is that processes can certainly be improved, thus reducing errors and eliminating the division between the various professional roles. Ultimately, it’s about understanding that both are equally important in achieving a unified goal” (I.03).

Positive Team Dynamics and Peer Support: It emphasizes the presence of a supportive, motivated team that values collaboration, continuous learning, and adaptation to new changes (I.03, I.04, I.08). “I feel supported by a team that is eager to work and learn—to work better and to learn something new—across all age groups, with different paces and rhythms depending on individual abilities and skills. However, everyone is motivated towards improvement and has a positive attitude towards this new implementation” (I.16). “We will need to adapt, and this is always a positive thing. Even if it will be difficult at the beginning and we make mistakes while trying to figure things out, I believe that, as a good team, we will still be able to find the right methods and timing. I am confident that our department has the qualities needed to succeed” (I.03).

Job Satisfaction: The potential for EHRs to provide a more comprehensive view of patient care activities was linked to increased job satisfaction. Being able to visualize the entirety of their work, rather than just individual tasks, could provide a greater sense of accomplishment and satisfaction with their roles (I.04, I.10). “We will all be much more personally accountable for the care provided. “There will be far fewer gray areas and uncertainties, such as “who did what” and “who was responsible in the morning, evening, or night”. This will increase traceability and, consequently, responsibilities. While increased traceability benefits the patient, if something goes wrong, it could potentially be used against the staff on duty. However, it’s fair if it protects the patient. If things are done properly and there is nothing to hide, we should be pleased with this change because it ultimately highlights and values our work” (I.07).

**Table 2:** Technostress creators and inhibitors.

Technostress Creators	Technostress Inhibitors
Initial Increased Workload and Time Demands	Increased Transparency and Accountability
Low Confidence in the Organization and Training Quality and Support	Reduction in Errors and Increased Accuracy
Device Equipment Issues	
User Experience of EHRs	
Low Interoperability and Paper Survivor	
Generational and Technological Proficiency Gaps	

Source: own elaboration.

## 4. Discussion

The findings of this study provide an understanding of how healthcare staff perceive the adoption of electronic health records (EHRs) and its impact on their well-being and workplace stress.

While EHR systems are frequently promoted as tools to streamline clinical workflows and reduce documentation errors, this study presents a more complex picture, revealing the dual nature of EHR implementation: it serves both as a facilitator of improved healthcare delivery and as a source of increased stress for staff.

One of the main themes that emerged was the perception of EHR adoption as a “double-edged sword”. On the positive side, participants acknowledged that the EHR system could streamline data entry, reduce documentation errors, and improve access to patient information. These benefits align with the existing literature that emphasizes the potential of EHRs to enhance patient safety and clinical outcomes [2]. However, the transition to EHRs was also associated with significant challenges, such as a steep learning curve, increased documentation time, disruptions to established workflows, and feelings of concern and anxiety. These findings are consistent with previous studies linking EHR adoption to technostress [25,26].

Participants expressed concerns that the increased time required for data entry could detract from direct patient care, potentially affecting job satisfaction and contributing to burnout [4]. This issue is particularly relevant in the context of Italy’s healthcare sector, where the rapid digital transformation driven by the National Recovery and Resilience Plan (PNRR) has placed additional demands on staff to quickly adapt to new systems.

The second major theme, “EHR’s Influence on Professional Dynamics”, highlighted the potential for EHR systems to reshape communication and collaboration within healthcare teams. Participants recognized that EHRs could enhance the quality of care by making essential patient data accessible to various healthcare providers. EHRs can offer relevant, timely, and current information that facilitates knowledge sharing and supports collaborative decision-making among multidisciplinary teams [27,28]. Others expressed concerns that might reduce direct, face-to-face interactions between staff members. This concern is supported by the existing literature, which suggests that dependence on electronic documentation can create barriers to effective communication and lead to a more fragmented approach to patient care [29].

The findings revealed generational differences in attitudes toward EHR adoption. Younger staff, more accustomed to using digital tools, generally viewed EHR implementation more favorably, while older staff expressed greater apprehension. These generational divides could potentially create power imbalances within teams, complicating the integration of EHR systems. To address these issues, healthcare organizations must prioritize comprehensive training programs and cultivate an environment of mutual support to bridge the gap between staff members with varying levels of technological proficiency [30].

Although many systematic reviews have examined the barriers and facilitators to EHR implementation, most studies have primarily focused on healthcare professionals, particularly physicians [10,31,32,33]. While comparisons of perspectives across different professional groups have been documented in the literature, these findings have not been synthesized. As interdisciplinary practice becomes more prevalent in the healthcare system, understanding and comparing the viewpoints of each user group is essential to ensure successful EHR implementation.

Many quantitative and qualitative studies have explored the impact of electronic health records (EHRs) on healthcare personnel, demonstrating that although these systems may initially decrease productivity, they can improve staff efficiency and the quality of care in the long run. For example, De Leon et al. found that, while the implementation of EHRs temporarily reduced productivity in an extensive primary care practice, providers showed significant increases in productivity once the system was coupled with pay-for-performance programs, allowing for a 1.7% monthly increase per provider [30]. Schumaker and Reganti highlighted that, although the adoption of EHRs faces barriers such as cost and data security issues, they can also improve the efficiency of healthcare delivery and enable better management of clinical information, mainly when implemented in cloud [31].

Bansler and From also noted that, although significant resources have been invested in the implementation of EHRs to improve productivity and patient safety, these benefits have not been fully realized due to deficiencies in system design, inappropriate configuration, and insufficient organizational implementation [32]. Likewise, Alami et al. suggested that EHRs can be a powerful tool for value-based healthcare systems. However, they emphasized the importance of aligning their implementation with improving healthcare professionals’ working conditions to maximize their benefits [33].

Studies such as that by Chen et al. found that, following the implementation of EHRs in an integrated care system, significant reductions in in-person visits were achieved while phone consultations and the use of secure email increased, reflecting improvements in operational efficiency and more patient-centered care [34]. Additionally, Kossman showed that, although EHRs may increase the time spent on documentation, nurses value their ability to improve access to information and patient safety [35].

Nagpal et al. highlighted that EHRs improve clinical documentation, record management, and efficiency in the healthcare system, although they face significant challenges related to high software costs and system security [35].

Overall, these studies highlight that, although EHRs present challenges during their implementation, their proper use can significantly improve efficiency and health outcomes.

Role-specific stressors also emerged as a critical finding. For physicians, the pressure to document patient care promptly led to concerns about documentation-related stress and potential legal liability. This stressor is especially relevant in scenarios where delays in documentation could result in legal consequences. Physicians expressed anxiety that retrospective scrutiny of records might lead to misinterpretation or malpractice accusations, heightening their stress.

For nurses, the assignment of non-nursing tasks emerged as a unique stressor. Nurses reported feeling burdened by additional administrative responsibilities, which detracted from their core clinical duties. This shift not only undermined their professional identity but also created a sense of role ambiguity and devaluation within the healthcare team. The findings underscore the need for healthcare organizations to clearly delineate roles and responsibilities to prevent nurses from being overburdened with non-clinical tasks. Despite these challenges, some nurses viewed the introduction of EHRs as an opportunity for greater recognition of their professional role within the team, potentially contributing to well-being. They expressed confidence in the support of a cohesive and younger team that is more receptive to digital innovations.

The challenges and experiences of EHR implementation in Italy offer important insights that could be applicable to other regions undergoing similar digital transitions. One key lesson is the importance of addressing technostress, which can arise from the increased demands and steep learning curves associated with digital tools. This is a more general problem for Italy. In fact, the DESI index identifies human capital as the weak point in Italy’s digitalization process: apparently, only 46% of the Italian population has basic IT skills [34,35]. Adequate training and support are critical to easing the transition for healthcare staff, as well-prepared teams are more likely to embrace EHR systems and experience less frustration and anxiety. Furthermore, fostering interprofessional collaboration is essential for successful EHR adoption. As shown in this study, the EHR system can facilitate better communication and teamwork when used effectively, but it can also disrupt established workflows if not carefully integrated into clinical practice. By focusing on these areas, healthcare systems in other regions can mitigate common barriers, streamline the adoption of new digital tools, and ultimately improve the outcomes of their digital transformation efforts.

### Study’s Limitations

This study presents several limitations that must be acknowledged. First, the sample size was small, limiting the ability to generalize the results to other contexts or populations. Moreover, the transferability of the findings could be improved, as this study focused on a single hospital unit. Likewise, there may be bias among the participants due to self-selection, as those with stronger opinions or greater interest in implementing electronic health records (EHRs) may have been more likely to participate. There is a possibility of bias on the part of the researchers despite efforts to mitigate this influence through reflexivity. Finally, workplace hierarchies may have influenced how participants expressed their perceptions, especially those in lower positions. Moreover, factors such as the department’s size and individual working hours could have affected the participants’ experiences and perceptions, which represents another limitation in the generalization of the findings.

## 5. Conclusions

EHR systems offer clear benefits, including better communication and information exchange, improving data accuracy, transparency and access, and reducing errors. The successful implementation of the system depends on addressing the challenges identified by staff.

In conclusion, this study highlights the complex and dual impact of EHR adoption on healthcare staff well-being and workplace stress. The qualitative approach offers a deep insight into the healthcare staff perspective. Findings disclose that it is essential to provide adequate training, ongoing technical support, and foster an inclusive environment that values the contributions of all team members, regardless of their technological proficiency. By doing so, healthcare organizations can maximize the benefits of EHR adoption while mitigating its potential drawbacks, ultimately promoting a healthier and more productive work environment for their staff.

### Future Lines of Research

For future studies, adopting a longitudinal approach to explore how participants’ perceptions of EHRs may change over time or under different circumstances would be valuable. This would offer a deeper insight into how experiences and perceptions evolve. Equally important is the need for a quantitative validation of the results. Without quantitative data to complement the qualitative findings of this study, it may not be easy to support broader claims or trends based on the data. A combination of qualitative and quantitative methods would strengthen the robustness and applicability of the conclusions, providing a more comprehensive view of the impact of EHRs on healthcare professionals.

For future research, it would also be valuable to explore how variables such as department size, hospital size, and working hours affect the implementation of EHRs on the well-being and work-related stress of healthcare staff. Comparative research between different departments and hospitals could provide a greater understanding of how these factors influence the adoption of EHRs and their impact on the mental health and working conditions of healthcare professionals. Likewise, future studies could benefit from a gender- and workplace-size-stratified analysis in more extensive and diverse samples, including multiple departments and hospitals. This would allow for a greater exploration of how these variables influence the effects of EHR implementation on healthcare staff’s well-being and work-related stress.

With this complement, future lines of research will be more comprehensive, integrating the need for a longitudinal approach, quantitative validation, and analysis of various variables in different hospital contexts.

## Figures and Tables

**Table 1:** Profession, experience, and gender.

Profession	Gender	Age Mean	Age Std	Experience Mean	Experience Std
Nurse	F	39.00	9.30	14.60	7.44
Nurse	M	34.50	3.54	6.00	1.41
Practitioner	F	56.60	6.66	28.20	6.65
Practitioner	M	59.00	9.64	32.00	7.94

This study includes a total of 16 participants: 8 nurses and 8 practitioners. Source: own elaboration.

## Data Availability

The data presented in this study are available from the corresponding author upon request.

## Appendix A. Interview Guide

(1) Can you briefly describe what the EHR system is?
(2) What are your general expectations regarding the upcoming implementation of the electronic health record (EHR) system?
(3) How do you think the transition to the EHR system will impact your daily work?
(4) What benefits do you expect from adopting the EHR system in your department?
(5) Do you have any concerns or fears about the introduction of the EHR system? If so, could you describe them?
(6) How do you think the introduction of the EHR system will affect your work efficiency and time management?
(7) Do you believe the EHR system will make managing clinical documentation easier or more complex?
(8) What are your expectations regarding the amount of time you will need to dedicate to documentation once the EHR system is implemented?
(9) Do you think the introduction of the EHR system might affect your psychological well-being at work? If so, how?
(10) What impact do you think the EHR system might have on your level of work-related stress? Why?
(11) Do you have concerns about a potential increase in stress or workload related to the use of the EHR system?
(12) What are your expectations regarding the training you will receive on the EHR system? Do you think it will be sufficient to prepare you?
(13) How do you assess the support provided by the organization in preparation for this transition?
(14) Do you feel adequately supported by your colleagues in view of this transition?
(15) What kind of support do you expect to receive during the EHR system introduction process to minimize any difficulties or stress?
(16) How could the introduction of the EHR system affect teamwork and collaboration with your colleagues?
(17) Do you expect the new system to influence communication among healthcare staff? If so, how?
(18) Do you believe the EHR system will improve transparency and information sharing within the team?
(19) Do you have any concerns regarding the introduction of the EHR system? If so, what are the main ones?
(20) What suggestions would you have to make the EHR implementation process smoother?
(21) What changes do you think the introduction of the EHR system will bring to your department or healthcare facility?
(22) Looking ahead, how do you imagine the EHR system will affect the well-being and stress levels of staff in the long term?
(23) Is there anything we haven’t discussed yet regarding the introduction of the EHR system that you consider important to highlight?
